# The Efficacy of Immunotherapy and Clinical Utility of Comprehensive Genomic Profiling in Adenoid Cystic Carcinoma of Head and Neck

**DOI:** 10.3390/medicina59122111

**Published:** 2023-12-01

**Authors:** Takahiro Naito, Rika Noji, Takuma Kugimoto, Takeshi Kuroshima, Hirofumi Tomioka, Shun Fujiwara, Mitsukuni Suenaga, Hiroyuki Harada, Yoshihito Kano

**Affiliations:** 1 Department of Clinical Oncology, Graduate School of Medical and Dental Sciences, Tokyo Medical and Dental University (TMDU), 1-5-45 Yushima, Bunkyo-Ku, Tokyo 113-8510, Japan; 2 Department of Oral and Maxillofacial Surgical Oncology, Division of Health Science, Graduate School of Medical and Dental Sciences, Tokyo Medical and Dental University (TMDU), 1-5-45 Yushima, Bunkyo-Ku, Tokyo 113-8510, Japan

**Keywords:** adenoid cystic carcinoma, head and neck, *MYB*, comprehensive genomic profiling test, immunotherapy, C-CAT database

## Abstract

*Background and Objectives*: Adenoid cystic carcinoma (ACC) of the head and neck is generally slow-growing but has a high potential for local recurrence and metastasis to distant organs. There is currently no standard pharmacological treatment for recurrent/metastatic (R/M) ACC, and there are cases in which immune checkpoint inhibitors (ICIs) are administered for ACC according to head and neck squamous cell carcinoma (HNSCC). However, the efficacy of ICIs for ACC remains unclear, and the predictive biomarkers need to be elucidated. *Materials and Methods*: The Center for Cancer Genomics and Advanced Therapeutics (C-CAT) database enabled the retrospective but nationwide analysis of 263 cases of ACC of the head and neck. Then, we examined and reported four cases of ACC that received ICIs and comprehensive genomic profiling (CGP) in our institution. *Results*: The C-CAT database revealed that 59 cases out of 263 received ICIs, and the best response was 8% of objective response rate (ORR) and 53% of disease control rate (DCR) (complete response, CR 3%, partial response, PR 5%, stable disease, SD 44%, progressive disease, PD 19%, not evaluated, NE 29%). The tumor mutational burden (TMB) in ACC was lower overall compared to HNSCC and could not be useful in predicting the efficacy of ICIs. Some cases with *MYB* structural variants showed the response to ICIs in the C-CAT database. A patient with *MYB* fusion/rearrangement variants in our institution showed long-term stable disease. *Conclusions*: ICI therapy is a potential treatment option, and the *MYB* structural variant might be a candidate for predictive biomarkers for immunotherapy in patients with R/M ACC.

## 1. Introduction

Adenoid cystic carcinoma (ACC) of the head and neck is an extremely rare type of cancer, accounting for about 1% of malignant head and neck tumors and 10% of malignant salivary gland tumors [1]. ACC is generally slow-growing but has a high potential for local recurrence and metastasis to distant organs; five-, ten-, and twenty-year survival rates for overall survival were 68%, 52%, and 28%, respectively [2,3]. Most patients with head and neck cancer (HNC) are treated with aggressive multidisciplinary approaches, including surgical resection, radiation therapy, and systemic chemotherapy, including molecular targeted agents or immune checkpoint inhibitors (ICIs). However, there is currently no standard systemic treatment for R/M ACC [4].

In recent years, ICI therapy has been developed and clinically applied to various cancers. Treatment with nivolumab and pembrolizumab, anti-programmed cell death-1 (PD-1) protein monoclonal antibodies, has been widely available for patients with recurrent or metastatic head and neck cancer (R/M HNC) [5]. The interaction between the PD-1 receptor and programmed cell death ligand-1 (PD-L1) inhibits T-cell immunological activities and is recognized as a representative mechanism of tumor immune evasion [6]. ICIs can enhance anti-tumor immune activity by blocking inhibitory signaling through the PD-1/PD-L1 pathway [7]. However, the response rate of ICI therapy has been shown to be 13–17% [8,9], and long-term response cases are limited. Therefore, the elucidation of biomarkers to predict ICI efficacy has been crucial for various types of solid tumors.

Recently, comprehensive genomic profiling (CGP) using next-generation sequencing (NGS) was developed in the clinical setting for the selection of promising molecularly targeted therapies. With the spread of CGP, cancer treatment has become increasingly personalized, and “precision medicine”, in which treatment is optimized for individuals according to their genetic mutations, is making progress [10]. Currently, biomarkers, such as microsatellite instability (MSI), the PD-L1 combined positive score (CPS), and tumor mutation burden (TMB), are used for selecting patients for clinical ICI therapy [11]. The population of MSI high is extremely low and could not be a universal marker for ICIs in patients with HNC [12]. It has been suggested that patients with TMB high had clinical benefits to ICIs, although PD-L1 CPS did not correlate with the efficacy of ICIs in real-world data in HNSCC [13]. There are cases in which ICIs are administered for ACC according to HNSCC. However, the efficacy of ICIs for ACC remains controversial [14] under the condition of the uninflamed and immunosuppressive tumor microenvironment in ACC [15,16].

This study examined the database of national genomic and clinical information established by the Center for Cancer Genomics and Advanced Therapy (C-CAT) based on the national policy to evaluate real-world data on genetic and clinical utility in R/M ACC of the head and neck. In addition, we investigated gene mutations using CGP in patients with R/M ACC who received ICIs according to HNSCC in our institution and evaluated the association between ICI response and predictive candidates, including TMB and genetic alterations in patients with ACC.

## 2. Materials and Methods

### 2.1. Patient Characterization on the C-CAT Database

We queried an anonymized database of genomic and clinical information on patients with cancer, collected from core hospitals using C-CAT. Cancer gene panel tests described in Section 2.3 were conducted at all designated hub hospitals in Japan. Gene profiling results were integrated into the C-CAT database at the National Cancer Center, Japan, and the data for each case from October 2021 could be utilized. The clinical data of C-CAT included age, sex, histology, type of cancer, treatment before and after the oncogene panel test, response to a drug, and type of CGP test. The study project in patients with R/M HNC was approved by the C-CAT Information Utilization Review Committee (proposal control number: CDU2022-021N).

### 2.2. Patient Population and Characterization at Tokyo Medical and Dental University (TMDU)

The clinical data, such as age, sex, performance status, and imaging evaluation before and after the systemic treatment for ACC, were collected. Computed tomography (CT) was performed at baseline and, thereafter, every 8–12 weeks until progression or treatment discontinuation. The 18-FDG-PET scan was performed once every year. We performed an oncogene panel test for R/M oral adenoid cystic carcinoma patients who were treated with immune checkpoint inhibitors as first-line therapy at our institution. In terms of informed consent, we obtained written consent from all patients for the use of genomic and clinical data for research purposes.

### 2.3. Comprehensive Genomic Profiling (CGP) Analysis

The C-CAT database currently includes the genomic information on all types of tumors from the Foundation One^®^ Companion Diagnostic (F1CDx; Foundation Medicine, Inc., Cambridge, MA, USA) test, Foundation One^®^ Liquid Companion Diagnostic (F1LCDx; Foundation Medicine, Inc.) test, and National Cancer Center (NCC) Oncopanel test. F1CDx and F1LCDx comprehensively analyzed 324 gene alterations, including substitutions, insertions, deletions, copy number changes, selective gene rearrangements, and calculated genomic signatures, such as MSI and TMB [17]. The NCC Oncopanel test examines mutations, amplifications, and homozygous deletions of the entire coding region of 127 genes of clinical or preclinical relevance, along with rearrangements [18]. The F1CDx test was used to obtain the genomic profiling in ACC of head and neck at our institution. The F1CDx assay used formalin-fixed paraffin-embedded (FFPE) tumor tissue samples obtained via biopsy or surgical operation, and the suitable tumor specimens for testing were selected by pathologists.

### 2.4. Expert Panel Discussion

Each result in the CGP test report was discussed at a molecular tumor board meeting by a multidisciplinary team. The board consisted of experts, such as clinical oncologists, pathologists, clinical geneticists, bioinformaticians, genomic researchers, and genetic counselors, and discussed issues, such as genetically informed treatment options and interpretation of somatic/genetic mutations. When a gene alteration is the target of a molecular targeted therapy, it is considered an actionable mutation. A drug is available for human use either as an antibody or as a small molecule compound with an IC50 concentration in the nanomolar range [19]. Evidence-level classification of gene aberrations was decided according to the Clinical Practice Guidance for NGS in Cancer Diagnosis and Treatment Edition 2.1 issued by three associations: the Japanese Society of Medical Oncology (JSMO), Japan Society of Clinical Oncology (JSCO), and Japanese Cancer Association (JCA) [20].

### 2.5. Efficacy Evaluation of ICI Therapy

We used anti-PD-1 inhibitors, such as nivolumab or pembrolizumab, and evaluated tumor response according to the Response Evaluation Criteria in Solid Tumors (RECIST) 1.1 criteria [21]. The objective response rate (ORR) was defined as the total number of patients who had complete response (CR) or partial response (PR). Disease control rate (DCR) was defined as the total number of patients with CR, PR, or stable disease (SD).

### 2.6. Biomarker Assessment

PD-L1 CPS score was evaluated in FFPE tumor samples using the PD-L1 immunohistochemistry (IHC) 22C3 pharmDx assay (Agilent Technologies, Santa Clara, CA, USA). TMB, defined as the total number of somatic mutations per million base pairs in the tumor genome, was measured using the CGP test [22]. TMB was divided into three groups: low (5 Muts/Mb or less), intermediate (6–9 Muts/Mb), and high scores (10 Muts/Mb or more), as described in a previous report [13].

## 3. Results

### 3.1. Comprehensive Patient Population and Genetic Characterization of CGP in C-CAT Data

**Figure 1:**
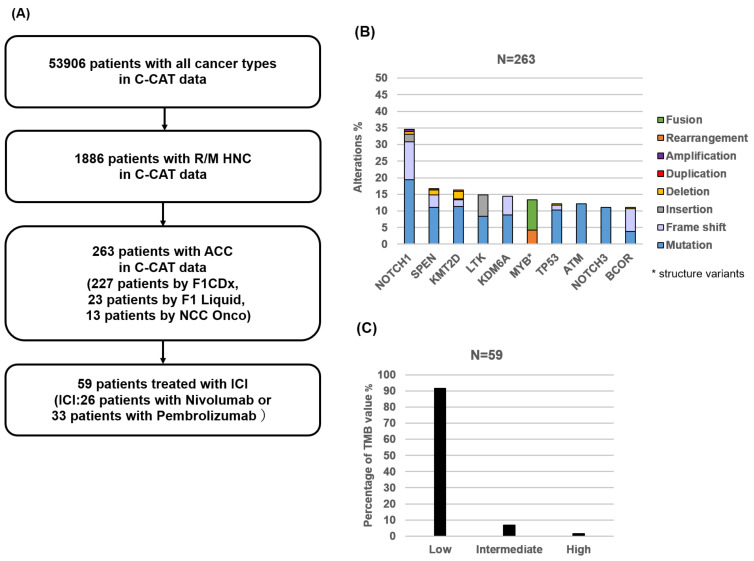
The scheme of total population and genetic information in ACC patients from CGP in the C-CAT database. (**A**) The scheme of the population from the C-CAT database using the F1(F1CDx or F1LCDx) test and NCC Oncopanel test. Information of 53,906 patients was registered in C-CAT, with a total of 263 patients diagnosed with R/M ACC of head and neck. (**B**) Percentage of the top 10 most frequent genes and variant types by histological type. Color coding indicates the variant type. (**C**) Percentage of TMB value in 59 patients who received ICI. TMB value is indicated as low (5 Muts/Mb or less), intermediate (6–9 Muts/Mb), and high (10 Muts/Mb or more).

Between June 2019 and June 2023, of the 53,906 patients enrolled in the C-CAT database, 1886 were classified with R/M HNC, of which 263 were categorized with ACC. Among them, 250 (F1 test) and 13 (NCC Oncopanel) patients were analyzed, respectively (Figure 1A). We found a total of 272 gene alterations and showed the top 10 most frequent genetic mutations detected in 263 ACC patients. The commonly mutated genes were *NOTCH1* (34%), followed by *SPEN* (17%), *KMT2D* (16%), *LTK* (15%), and *KDM6A* (14%), which is mainly consistent with a previous large study [23]. *MYB* structure variants were about 14% (Figure 1B). 

### 3.2. Outcomes of ICI Therapy in C-CAT Data

Of the 263 patients with R/M ACC, 59 (22%) received immune checkpoint inhibitors, of which 26 were treated with nivolumab and 33 with pembrolizumab. First, we examined the treatment efficacy of 59 ACC patients. Of these, seventeen (29%) cases were not evaluable (NE). Two (3%) patients experienced CR, three (5%) had PR, twenty-six (44%) had SD, and eleven (19%) had PD; the ORR was 8%, and the DCR was 53%. The patients were divided into three groups (low, intermediate, and high) according to TMB values to evaluate the differences in ICI treatment efficacy, as described in a previous report [13]. Of 59 ACC patients, 54 (92%) were in the low TMB, 4 (7%) were in the intermediate, and 1 (2%) was in the high group (Figure 1C). In the TMB-low group, the best response to ICIs was CR in 2/54 (4%), PR in 3/54 (6%), and SD in 23/54 (43%) patients, and, therefore, the ORR was 9% and the DCR was 52%. The TMB-intermediate group showed SD in 2/4 (50%) patients and DCR of 50%. One patient in the TMB-high group was SD (Table 1A). The median TMB was 2 Muts/Mb (range, 0–11). The median age of the patients with ACC was 61 years (range: 34–77 years), and 54% were female. In terms of results by gender, the ORR was 7% and the DCR was 56% for males and 9% and 50% for females, respectively (Table 1B).

Furthermore, we compared the patients who received ICIs as first-line therapy with second-line or later-line therapy to eliminate the ambiguous difference between therapy lines. Of the 59 patients, 28 received ICIs as the first-line therapy and 31 received ICIs as the second-line or later-line therapy. In the 28 patients who received ICIs as first-line therapy, 1 (4%) patient experienced CR, 1 (4%) had PR, 8 (29%) had SD, 7 (25%) had PD, and 11 (39%) were NE; the ORR was 7% and the DCR was 36%. Of 28 patients, 25 (89%) were in the low-TMB group, 3 (11%) in the intermediate group, and no patients were in the high group. The best response to ICIs in the TMB-low group was CR in 1/25 (4%), PR in 1/25 (4%), and SD in 6/25 (24%) patients, and, therefore, the ORR was 8% and the DCR was 32%. The TMB-intermediate group showed SD in 2/3 (66%) patients and a DCR of 67% (Table 2A). In the 31 patients who received ICIs as the second-line or later-line therapy, 1 (3%) patient experienced CR, 2 (6%) had PR, 18 (58%) had SD, 4 (13%) had PD, and 6 (19%) were NE; the ORR was 10% and the DCR was 68%. Of 31 patients, 29 (94%) were in the low-TMB group, 1 (3%) in the intermediate group, and 1 (3%) was in the high group. The best response to ICIs in the TMB-low group was CR in 1/29 (3%), PR in 2/29 (7%), and SD in 17/29 (59%) patients, and, therefore, the ORR was 11% and the DCR was 69%. One patient in the TMB-intermediate group was PD, and one patient in the TMB-high group was SD (Table 2B). Thus, the patients who received ICIs as first-line or second/later-line therapy had a similar ORR, and TMB could not be a predictive marker for ICI therapy due to the extremely low number of patients with ACC of the head and neck.

Next, we examined the treatment efficacy in ACC patients with *NOTCH1*, *KDM6A*, *BRAF* mutations, and *MYB* structural variants, as these genes were commonly observed and associated with poorer prognosis in ACC [23,24]. *NOTCH1*, *BRAF* mutations, and *MYB* structural variants were found in thirteen, four, five, and five patients, respectively. In *NOTCH1* and *BRAF* mutation cases, the ORR was 0% and the DCR was about 50%. In *KDM6A* mutation cases, the ORR was 0% and the DCR was 20%. In the cases with *MYB* structure variants, one (20%) patient experienced CR, and one (20%) had PR; both ORR and DCR were 40% (Table 3). The five cases that were CR or PR are shown in Table 4. The median TMB was 1.26 Muts/Mb for CR and PR cases that did not correlate with treatment response.

### 3.3. Outcomes of ICI Therapy and Sequencing Results in TMDU

Between 26 February 2020 and 31 May 2023, four patients with R/M ACC who were treated with immune checkpoint inhibitors as first-line therapy underwent CGP with an F1 test at TMDU. We examined the treatment response of four ACC patients. All patients expressed more than 1% of PD-L1 CPS, which allowed for the administration of ICIs. All patients were treated with pembrolizumab; of these, three patients experienced SD, and one had PD. TMB values were ≤5 Muts/Mb in all patients (Table 5A).

We evaluated the genetic mutations detected in four R/M ACC patients who were treated for ICI at our institution. The commonly mutated genes were *NOTCH1*, *TP53*, and *TERT* promoter. *MYB* rearrangement and *MYB-NFIB* fusion were found in the same case (Table 5B). Of the four patients, one patient with *MYB* structure variants who had a long-term stable disease is shown below.

### 3.4. Case Presentation (Case No.3 in Table 5A,B)

**Figure 2:**
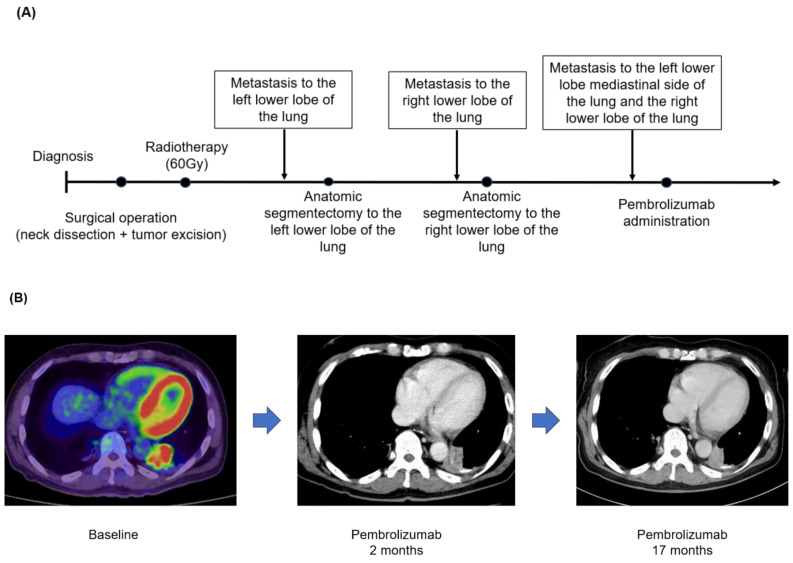
(**A**) Clinical course. (**B**) FDG-PET or CT scan before therapy with pembrolizumab, 2 months after treatment, and 17 months since he initiated the ICI therapy. The red color means FDG uptake.

A 63-year-old man was diagnosed with a submandibular gland tumor and underwent a submandibular adenectomy in December 2012. The intraoperative frozen section revealed ACC, so concurrent neck dissection (level I-IIA) was performed. Thereafter, he underwent adjuvant radiotherapy (60 Gy) in January 2013. In February 2020, a CT scan showed metastasis to the left lower lobe of the lung, and he underwent an anatomic segmentectomy. Histopathological examination identified a metastatic tumor consistent with ACC of the submandibular gland tumor. In addition, in October 2020, a CT scan confirmed metastasis to the right lower lobe of the lung, an anatomic segmentectomy was performed, and a histopathologic examination identified a metastatic tumor consistent with ACC of the primary tumor. Furthermore, the 18-FDG-PET scan showed new metastasis to both side lobes of the lung, and the NGS panel using F1CDx revealed that the TMB was 1 Muts/Mb and showed mutated genes, including *BRAF*, *NOTCH1*, and *MYB* structural variants (Table 5B). Pembrolizumab was started in January 2022 (Figure 2A), and two months after administration, a CT scan showed a significant reduction in the target lesions. Seventeen months have passed since pembrolizumab’s first administration, and the target lesions were maintained without new recurrence and metastases (Figure 2B).

## 4. Discussion

We herein conducted a retrospective study of the response to ICI therapy for patients with ACC of the head and neck based on CGP from the nationwide database and institutional cohort. Information on cancer gene panel tests conducted at all designated hospitals in Japan was, in principle, integrated into the C-CAT database. Thus, this database sheds light on the molecular biology or genetic findings in a rare type of cancer such as ACC.

First, we examined the clinical analysis of CGP in 263 ACCs of the head and neck in C-CAT data. The commonly mutated genes were *NOTCH1* (34%), followed by *SPEN* (17%), *KMT2D* (16%), *LTK* (15%), *KDM6A* (14%), and *MYB* structure variants (14%). The NOTCH signaling pathway plays an important role in regulating cell proliferation and survival. *NOTCH1* mutations in ACC occur in advanced-stage disease, distinct metastatic patterns, and poor prognosis [24]. *KDM6A* is important for the differentiation of embryonic stem cells and the development of various tissues and functions as a tumor suppressor [25]. *NOTCH1* and *KDM6A* mutations have been reported to be associated with poorer prognosis compared to *NOTCH1* and *KDM6A* wild-type patients, respectively [23].

*MYB* structure variants were called at a frequency of 14% from C-CAT data analysis, which was lower compared to the previous reports of 65–82% of ACC in the head and neck [26,27]. One reason is that *MYB* structure variants are not included in the list of NCC Oncopanel, and the other is that NGS panel tests (F1 and NCC Oncopanel) are exclusively performed using the DNA sequence, resulting in lower detection of structural variants, including rearrangement or fusion. MYB is a nuclear transcription factor and frequently occurs in the chromosome t(6;9) translocation with a transcription factor, NFIB, leading to the MYB-NFIB fusion that is considered to be a genetic hallmark of ACC [28]. MYB also increased metastasis by regulating ICAM1, VEGFA, MMP7, MMP9, and EMT-related markers, such as E-cadherin, vimentin, N-cadherin, and alpha-SMA. In addition, it was determined that MYB was closely associated with lung metastasis in patients with salivary adenoid cystic carcinoma through a xenograft mouse model [29] while it has been reported that no significant prognostic differences were observed between MYB-positive and MYB-negative ACC patients in the head and neck [30]. Importantly, the tyrosine kinase inhibitor (TKI) axitinib showed a trend toward superior progression-free survival (PFS) in ACC patients with MYB/NFIB rearrangements [31].

Second, we analyzed the efficacy of 59 ACC patients who were treated with ICIs. Due to its molecular and histological characteristics, such as lower immunogenicity, low TMB, fewer tumor-infiltrating lymphocytes, fewer dendritic cells, and lower levels of PD-1+ and CTLA4+ cells [15,32,33], the efficacy of ICI therapy has been limited, although promising data were also reported in R/M ACC [14,34]. In the C-CAT cohort, two (3%) patients experienced CR, three (5%) had PR, twenty-six (44%) had SD, and eleven (19%) had PD; the ORR was 8% and the DCR was 53%. It has been suggested that a higher value of TMB shows a positive association with higher response rates to ICI therapy in solid tumors. It was found that the median TMB was 2 Muts/Mb (range, 0–11) in ACC of the head and neck, while the TMB median was 5 Muts/Mb (range, 0–34) in our previous report in HNSCC [13]. There was only one case in the TMB-high group with SD. Therefore, there are only a few cases in patients with TMB high in R/M ACC, and TMB could not be a useful predictive marker.

Next, the efficacy of ICIs was analyzed with genetic mutations, such as *NOTCH1*, *KDM6A*, *BRAF*, and *MYB*. There are no response cases of ICIs with *NOTCH1*, *KDM6A*, or *BRAF* mutations, while *MYB* structural alteration showed 40% of ORR, suggesting *MYB* might be a candidate marker for a better prognosis for ICIs. Then, we examined the treatment response to ICIs and the clinical analysis of CGP in our institution. We found that the case with *MYB* structure variants had a long-term response, even with co-mutation of *NOTCH1* and *BRAF* activation, suggesting that *MYB* alteration might have a strong effect on ICI efficacy. Intriguingly, a recent study showed that MYB is essential for the development of precursors of exhausted T cells, and the response to PD-1 checkpoint inhibition depends on MYB expression [35], which is consistent with our results. Furthermore, very recently, the addition of the PD-L1 inhibitor avelumab to axitinib has been shown to be beneficial in ACC of the head and neck [36], leading to its inclusion as a therapeutic option for ACC in the National Comprehensive Cancer Network guidelines.

To the best of our knowledge, this report could be the first in a real-world setting as there has been no report on the response of ICIs in ACC of the head and neck based on a comprehensive genomic profile. In C-CAT data, there were no ICI responses in patients with *NOTCH1*, *KDM6A*, or *BRAF* mutations, but there were response cases with *MYB* structure variants. In our institution, one patient with *MYB* structure variants had a long-term response, despite the presence of a *NOTCH1* mutation. This result is expected to help predict the efficacy of ICI therapy in patients with R/M ACC of the head and neck.

There are a few limitations in this study. First, although the C-CAT database provided comprehensive results of genomic and clinical information regarding the response to therapies, such as the ORR or DCR, overall survival (OS) or progression-free survival (PFS) is not currently available. Therefore, we need to evaluate the survival rate in our institute; however, the sample size was very small so we could not perform any statistical analysis here. Second, this study was conducted domestically and retrospectively. In the future, further investigation should be prospectively conducted with an international multicentric cohort, including a larger sample size with *MYB* structural variants to confirm the results of this study.

## 5. Conclusions

In this study, we evaluated the clinical utility of CGP and responses to ICIs in head and neck ACCs using a nationwide database and institutional cohort. We found commonly mutated genes, such as *NOTCH1* (34%), followed by *SPEN* (17%), *KMT2D* (16%), *LTK* (15%), *KDM6A* (14%), and *MYB* structure variants (14%). Interestingly, the population frequency of MYB structural variants was lower compared to previous reports. Notably, the C-CAT database revealed that ICI therapy did not respond in ACC patients with *NOTCH1*, *KDM6A*, or *BRAF* mutations, but there were response cases with *MYB* structure variants. Furthermore, we showed a case of long-term response to ICIs in our institution with *MYB* structural variants. ICI therapy could be a potential treatment option; however, the TMB value in ACC was relatively lower overall compared to HNSCC and could not be universally useful in predicting the efficacy of ICIs. The *MYB* structural variant might be a candidate for predictive biomarkers for immunotherapy in patients with R/M ACC.

## Figures and Tables

**Table 1:** (A) Response to ICIs in 59 ACC patients. (B) Outcomes by gender and age (CR; complete response, PR; partial response, SD; stable disease, PD; progressive disease, NE; not evaluated, ORR; objective response rate, DCR; disease control rate, TMB; tumor mutational burden).

**(A)**										
		n (%)	Best response					ORR		DCR
			CR	PR	SD	PD	NE			
All patients		59 (100)	2 (3)	3 (5)	26 (44)	11 (19)	17 (29)	5 (8)		31 (53)
TMB		59 (100)								
Low (≤5)		54 (92)	2 (4)	3 (6)	23 (43)	10 (19)	16 (31)	5/54 (9)		28/54 (52)
Intermediate (5–10)		4 (7)	0 (0)	0 (0)	2 (50)	1 (25)	1 (25)	0/4 (0)		2/4 (50)
High (>10)		1 (2)	0 (0)	0 (0)	1 (100)	0 (0)	0 (0)	0/1 (0)		1/1 (100)
**(B)**										
	n (%)		Age (y)				Outcome (%)			
			Median		Range		ORR		DCR	
All patients	59 (100)		61		34–77		5/59 (8)		31/59 (53)	
Male	27 (46)		60		36–77		2/27 (7)		15/27 (56)	
Female	32 (54)		62		34–77		3/32 (9)		16/32 (50)	

**Table 2:** (A) Twenty-eight patients received ICIs as first-line therapy. (B) Thirty-one patients received ICIs as second-line or late therapy.

**(A)**								
	n (%)	Best response					ORR	DCR
		CR	PR	SD	PD	NE		
All patients	28 (100)	1 (4)	1 (4)	8 (29)	7 (25)	11 (39)	2 (7)	10 (36)
TMB	28 (100)							
Low (≤5)	25 (89)	1 (4)	1 (4)	6 (24)	7 (28)	10 (40)	2/25 (8)	8/25 (32)
Intermediate (5–10)	3 (11)	0 (0)	0 (0)	2 (67)	0 (0)	1 (33)	0/0 (0)	2/3 (67)
High (>10)	0 (0)	0 (0)	0 (0)	0 (0)	0 (0)	0 (0)	0/0 (0)	0/0 (0)
**(B)**								
	n (%)	Best response					ORR	DCR
		CR	PR	SD	PD	NE		
All patients	31 (100)	1 (3)	2 (6)	18 (58)	4 (13)	6 (19)	3 (10)	21 (68)
TMB	31 (100)							
Low (≤5)	29 (90)	1 (3)	2 (7)	17 (59)	3 (10)	6 (21)	3/29 (11)	20/29 (69)
Intermediate (5–10)	1 (7)	0 (0)	0 (0)	0 (0)	1 (100)	0 (0)	0/1 (0)	0/1 (0)
High (>10)	1 (4)	0 (0)	0 (0)	1 (100)	0 (0)	0 (0)	0/1 (0)	1/1 (100)

**Table 3:** Response to ICIs for each gene mutation.

	n	Best Response (%)					ORR	DCR
		CR	PR	SD	PD	NE		
NOTCH1	13	0 (0)	0 (0)	6 (46)	3 (23)	4 (30)	0/13 (0)	6/13 (46)
BRAF	4	0 (0)	0 (0)	2 (50)	0 (0)	2 (50)	0/4 (0)	2/4 (50)
KDM6A	5	0 (0)	0 (0)	1 (20)	1 (20)	3 (60)	0/5 (0)	1/5 (20)
MYB *	5	1 (20)	1 (20)	0 (0)	2 (40)	1 (20)	2/5 (40)	2/5 (40)

* structure variants.

**Table 4:** Five patients who had CR or PR in response to ICIs.

Case No.	Age(yr)/Gender	Tumor Mutational Burden	Immune Checkpoint Inhibitor	Treatment Line	Best Response
1	53/F	5.04 mut/Mb	Pembrolizumab	1st line	CR
2	55/M	0 mut/Mb	Nivolumab	2nd line	CR
3	63/M	2 mut/Mb	Pembrolizumab	1st line	PR
4	62/F	1.26 mut/Mb	Pembrolizumab	2nd line	PR
5	34/F	1 mut/Mb	Nivolumab	3rd line	PR

**Table 5:** (A) Four ACC patients received ICIs at our institution. (B) Sequencing results.

**(A)**						
Case No.	Age(yr)/Gender	Primary site	Tumor Mutational Burden	Combined positive score	Immune checkpoint inhibitor	Best Response
1	57/M	Mandibular gingival	1.26 mut/Mb	2–3%	Pembrolizumab	SD
2	61/M	Palate	1.26 mut/Mb	1–3%	Pembrolizumab	PD
3	70/M	Submandibular gland	1.26 mut/Mb	1–3%	Pembrolizumab	SD
4	49/F	Submandibular gland	2.52 mut/Mb	1–5%	Pembrolizumab	SD
**(B)**						
Case No.	Genomic Findings					
1	NOTCH1 S990fs*34,H1544fs*66 U2AF1 S34F					
2	TP53 R273C					
3	BRAF G469A EP300 R202* MLL2 G61fs*69 MYB-NFIB fusion MYB rearrangement intron 14 NOTCH1 H2428fs*6 TERT promoter -124C>T TP53 C176W-subclonal					
4	RPTOR D1101Y TERT promoter -124C>T CEBPA P222T CARD11 L601P,T670M SPEN R3403H					

* a translation stop codon.

## Data Availability

Restrictions apply to the availability of these data. Data was obtained from C-CAT and are available from the authors with the permission of C-CAT.

## References

[1] Atallah S., Casiraghi O., Fakhry N., Wassef M., Uro-Coste E., Espitalier F., Sudaka A., Kaminsky M.C., Dakpe S., Digue L. (2020). A prospective multicentre REFCOR study of 470 cases of head and neck Adenoid cystic carcinoma: Epidemiology and prognostic factors. Eur. J. Cancer.

[2] Jeong I.S., Roh J.L., Cho K.J., Choi S.H., Nam S.Y., Kim S.Y. (2020). Risk factors for survival and distant metastasis in 125 patients with head and neck adenoid cystic carcinoma undergoing primary surgery. J. Cancer Res. Clin. Oncol..

[3] Van Weert S., Bloemena E., van der Waal I., de Bree R., Rietveld D.H., Kuik J.D., Leemans C.R. (2013). Adenoid cystic carcinoma of the head and neck: A single-center analysis of 105 consecutive cases over a 30-year period. Oral Oncol..

[4] Lorini L., Ardighieri L., Bozzola A., Romani C., Bignotti E., Buglione M., Guerini A., Lombardi D., Deganello A., Tomasoni M. (2021). Prognosis and management of recurrent and/or metastatic head and neck adenoid cystic carcinoma. Oral Oncol..

[5] Adelstein D., Gillison M.L., Pfister D.G., Spencer S., Adkins D., Brizel D.M., Burtness B., Busse P.M., Caudell J.J., Cmelak A.J. (2017). NCCN Guidelines Insights: Head and Neck Cancers, Version 2.2017. J. Natl. Compr. Cancer Netw..

[6] Ferris R.L. (2015). Immunology and Immunotherapy of Head and Neck Cancer. J. Clin. Oncol..

[7] Cohen E.E.W., Bell R.B., Bifulco C.B., Burtness B., Gillison M.L., Harrington K.J., Le Q.T., Lee N.Y., Leidner R., Lewis R.L. (2019). The Society for Immunotherapy of Cancer consensus statement on immunotherapy for the treatment of squamous cell carcinoma of the head and neck (HNSCC). J. Immunother. Cancer.

[8] Burtness B., Harrington K.J., Greil R., Soulieres D., Tahara M., de Castro G., Psyrri A., Baste N., Neupane P., Bratland A. (2019). Pembrolizumab alone or with chemotherapy versus cetuximab with chemotherapy for recurrent or metastatic squamous cell carcinoma of the head and neck (KEYNOTE-048): A randomised, open-label, phase 3 study. Lancet.

[9] Ferris R.L., Blumenschein G., Fayette J., Guigay J., Colevas A.D., Licitra L., Harrington K., Kasper S., Vokes E.E., Even C. (2016). Nivolumab for Recurrent Squamous-Cell Carcinoma of the Head and Neck. N. Engl. J. Med..

[10] Haendel M.A., Chute C.G., Robinson P.N. (2018). Classification, Ontology, and Precision Medicine. N. Engl. J. Med..

[11] Schlauch D., Fu X., Jones S.F., Burris H.A., Spigel D.R., Reeves J., McKenzie A.J. (2021). Tumor-Specific and Tumor-Agnostic Molecular Signatures Associated With Response to Immune Checkpoint Inhibitors. JCO Precis. Oncol..

[12] Blons H., Cabelguenne A., Carnot F., Laccourreye O., de Waziers I., Hamelin R., Brasnu D., Beaune P., Laurent-Puig P. (1999). Microsatellite analysis and response to chemotherapy in head-and-neck squamous-cell carcinoma. Int. J. Cancer.

[13] Noji R., Tohyama K., Kugimoto T., Kuroshima T., Hirai H., Tomioka H., Michi Y., Tasaki A., Ohno K., Ariizumi Y. (2022). Comprehensive Genomic Profiling Reveals Clinical Associations in Response to Immune Therapy in Head and Neck Cancer. Cancers.

[14] Tchekmedyian V., Sherman E.J., Dunn L., Fetten J.V., Michel L.S., Kriplani A., Morris L., Ostrovnaya I., Katabi N., Haque S. (2019). A phase II trial cohort of nivolumab plus ipilimumab in patients (Pts) with recurrent/metastatic adenoid cystic carcinoma (R/M ACC). J. Clin. Oncol..

[15] Mosconi C., de Arruda J.A.A., de Farias A.C.R., Oliveira G.A.Q., de Paula H.M., Fonseca F.P., Mesquita R.A., Silva T.A., Mendonça E.F., Batista A.C. (2019). Immune microenvironment and evasion mechanisms in adenoid cystic carcinomas of salivary glands. Oral Oncol..

[16] Guazzo E., Cooper C., Wilkinson L., Feng S., King B., Simpson F., Porceddu S., Panizza B., Coward J.I.G. (2021). Therapeutic implications of immune-profiling and EGFR expression in salivary gland carcinoma. Head Neck.

[17] Frampton G.M., Fichtenholtz A., Otto G.A., Wang K., Downing S.R., He J., Schnall-Levin M., White J., Sanford E.M., An P. (2013). Development and validation of a clinical cancer genomic profiling test based on massively parallel DNA sequencing. Nat. Biotechnol..

[18] Sunami K., Ichikawa H., Kubo T., Kato M., Fujiwara Y., Shimomura A., Koyama T., Kakishima H., Kitami M., Matsushita H. (2019). Feasibility and utility of a panel testing for 114 cancer-associated genes in a clinical setting: A hospital-based study. Cancer Sci..

[19] Matsudera S., Kano Y., Aoyagi Y., Tohyama K., Takahashi K., Kumaki Y., Mitsumura T., Kimura K., Onishi I., Takemoto A. (2021). A Pilot Study Analyzing the Clinical Utility of Comprehensive Genomic Profiling Using Plasma Cell-Free DNA for Solid Tumor Patients in Japan (PROFILE Study). Ann. Surg. Oncol..

[20] Naito Y., Aburatani H., Amano T., Baba E., Furukawa T., Hayashida T., Hiyama E., Ikeda S., Kanai M., Kato M. (2021). Clinical practice guidance for next-generation sequencing in cancer diagnosis and treatment (edition 2.1). Int. J. Clin. Oncol..

[21] Eisenhauer E.A., Therasse P., Bogaerts J., Schwartz L.H., Sargent D., Ford R., Dancey J., Arbuck S., Gwyther S., Mooney M. (2009). New response evaluation criteria in solid tumours: Revised RECIST guideline (version 1.1). Eur. J. Cancer.

[22] Büttner R., Longshore J.W., López-Ríos F., Merkelbach-Bruse S., Normanno N., Rouleau E., Penault-Llorca F. (2019). Implementing TMB measurement in clinical practice: Considerations on assay requirements. ESMO Open.

[23] Ho A.S., Ochoa A., Jayakumaran G., Zehir A., Valero Mayor C., Tepe J., Makarov V., Dalin M.G., He J., Bailey M. (2019). Genetic hallmarks of recurrent/metastatic adenoid cystic carcinoma. J. Clin. Investig..

[24] Ferrarotto R., Mitani Y., Diao L., Guijarro I., Wang J., Zweidler-McKay P., Bell D., William W.N., Glisson B.S., Wick M.J. (2017). Activating NOTCH1 Mutations Define a Distinct Subgroup of Patients With Adenoid Cystic Carcinoma Who Have Poor Prognosis, Propensity to Bone and Liver Metastasis, and Potential Responsiveness to Notch1 Inhibitors. J. Clin. Oncol..

[25] Tran N., Broun A., Ge K. (2020). Lysine Demethylase KDM6A in Differentiation, Development, and Cancer. Mol. Cell Biol..

[26] Brill L.B., Kanner W.A., Fehr A., Andrén Y., Moskaluk C.A., Löning T., Stenman G., Frierson H.F. (2011). Analysis of MYB expression and MYB-NFIB gene fusions in adenoid cystic carcinoma and other salivary neoplasms. Mod. Pathol..

[27] Rettig E.M., Tan M., Ling S., Yonescu R., Bishop J.A., Fakhry C., Ha P.K. (2015). MYB rearrangement and clinicopathologic characteristics in head and neck adenoid cystic carcinoma. Laryngoscope.

[28] Humtsoe J.O., Kim H.S., Jones L., Cevallos J., Boileau P., Kuo F., Morris L.G.T., Ha P. (2022). Development and Characterization of MYB-NFIB Fusion Expression in Adenoid Cystic Carcinoma. Cancers.

[29] Xu L.H., Zhao F., Yang W.W., Chen C.W., Du Z.H., Fu M., Ge X.Y., Li S.L. (2019). MYB promotes the growth and metastasis of salivary adenoid cystic carcinoma. Int. J. Oncol..

[30] Liu X., Chen D., Lao X., Liang Y. (2019). The value of MYB as a prognostic marker for adenoid cystic carcinoma: Meta-analysis. Head Neck.

[31] Ho A.L., Dunn L., Sherman E.J., Fury M.G., Baxi S.S., Chandramohan R., Dogan S., Morris L.G., Cullen G.D., Haque S. (2016). A phase II study of axitinib (AG-013736) in patients with incurable adenoid cystic carcinoma. Ann. Oncol..

[32] Ross J.S., Gay L.M., Wang K., Vergilio J.A., Suh J., Ramkissoon S., Somerset H., Johnson J.M., Russell J., Ali S. (2017). Comprehensive genomic profiles of metastatic and relapsed salivary gland carcinomas are associated with tumor type and reveal new routes to targeted therapies. Ann. Oncol..

[33] Witte H.M., Gebauer N., Lappöhn D., Umathum V.G., Riecke A., Arndt A., Steinestel K. (2020). Prognostic Impact of PD-L1 Expression in Malignant Salivary Gland Tumors as Assessed by Established Scoring Criteria: Tumor Proportion Score (TPS), Combined Positivity Score (CPS), and Immune Cell (IC) Infiltrate. Cancers.

[34] Cohen R.B., Delord J.P., Doi T., Piha-Paul S.A., Liu S.V., Gilbert J., Algazi A.P., Damian S., Hong R.L., Le Tourneau C. (2018). Pembrolizumab for the Treatment of Advanced Salivary Gland Carcinoma: Findings of the Phase 1b KEYNOTE-028 Study. Am. J. Clin. Oncol..

[35] Tsui C., Kretschmer L., Rapelius S., Gabriel S.S., Chisanga D., Knöpper K., Utzschneider D.T., Nüssing S., Liao Y., Mason T. (2022). MYB orchestrates T cell exhaustion and response to checkpoint inhibition. Nature.

[36] Ferrarotto R., Sousa L.G., Feng L., Mott F., Blumenschein G., Altan M., Bell D., Bonini F., Li K., Marques-Piubelli M.L. (2023). Phase II Clinical Trial of Axitinib and Avelumab in Patients With Recurrent/Metastatic Adenoid Cystic Carcinoma. J. Clin. Oncol..

